# Equine Allogeneic Chondrogenic Induced Mesenchymal Stem Cells Are an Effective Treatment for Degenerative Joint Disease in Horses

**DOI:** 10.1089/scd.2018.0061

**Published:** 2019-03-08

**Authors:** Sarah Y. Broeckx, Bert Seys, Marc Suls, Aurélie Vandenberghe, Tom Mariën, Edouard Adriaensen, Jeroen Declercq, Lore Van Hecke, Gabriele Braun, Klaus Hellmann, Jan H. Spaas

**Affiliations:** ^1^ Global Stem cell Technology NV, Anacura Group, Evergem, Belgium.; ^2^ Equine Veterinary Practice Dr Suls, SP Weert, the Netherlands.; ^3^ Equine Veterinary Service Adriaensen-Vandenberghe, Waasmunster, Belgium.; ^4^ Equitom Equine Hospital, Meldert, Belgium.; ^5^ Veterinary Practitioner, Oudenaarde, Belgium.; ^6^ Klifovet AG, Munich, Germany.

**Keywords:** allogeneic, MSC, arthrosis, horse, GCP, field trial

## Abstract

Degenerative joint disease is one of the main causes of equine early retirement from pleasure riding or a performance career. The disease is initially triggered by an abnormal loading of normal cartilage or a normal loading of abnormal cartilage. This primary insult is accompanied with joint inflammation, which leads to further progressive degeneration of the articular cartilage and changes in the surrounding tissues. Therefore, in search for an effective treatment, 75 adult horses with early signs of degenerative fetlock joint disease were enrolled in a randomized, multicenter, double-blinded, and placebo-controlled study. Fifty animals were injected intra-articularly with the investigational veterinary product (IVP) consisting of allogeneic chondrogenic induced mesenchymal stem cells (ciMSCs) with equine allogeneic plasma, and 25 horses were injected with 0.9% NaCl (saline) control product. From week 3 to 18 after treatment, lameness scores (*P* < 0.001), flexion test responses (*P* < 0.034), and joint effusion scores (*P* < 0.001) were remarkably superior in IVP-treated horses. Besides nasal discharge in both treatment groups, no adverse events were observed during the entire study period. On long-term follow-up (1 year), significantly more investigational product-treated horses were working at training level or were returned to their previous level of work (*P* < 0.001).

## Introduction

Just like in human medicine, degenerative joint disease is a well-known and common problem in equine medicine. The disease starts with an injury or pathology in the soft tissues, subchondral bone, or articular cartilage of the joint or with a combination of the above. This initial trigger leads to progressive degradation of the articular cartilage together with changes in the bone and the surrounding soft tissues [1]. Whatever the primary cause may be, joint inflammation or, more specifically, synovitis is an important aggravator or mediator of degenerative joint disease. Indeed, synovitis leads to the production of proinflammatory cytokines and matrix-degrading enzymes, contributing to the disease development [2,3]. Moreover, synovitis causes pain and joint effusion, which contributes to joint (micro-)instability.

Approximately 25% of horses are affected with some stage of degenerative joint disease at some point of their lifetime, be it an early stage mainly characterized by an inflammatory component and altered cartilage metabolism or a more chronic stage characterized by bony changes with intermittent inflammatory flares and severe cartilage destruction [4,5]. Considering that there are ∼5.7 million horses registered in official databases in Europe [6] and 9.2 million in the United States [7], and that, according to an official European market study from Euromonitor, ∼25% of injured horses visit a veterinarian regularly, 356,000 European horses and 575,000 American horses that visit a veterinarian suffer from osteoarthritis at a certain point in their life.

Currently, in both human and veterinary medicine, different treatments are available to ease the pain and increase the patient's comfort. Similar to human medicine, the most frequently used medicinal products in equine practice for early-stage joint inflammation are nonsteroidal anti-inflammatory drugs (NSAIDs), corticosteroids, glucosamine, chondroitin sulfate, and hyaluronic acid [8–10].

NSAIDs moderately reduce the pain and inflammation associated with degenerative joint disease. However, they require daily administration and gastrointestinal and kidney problems are well-known side effects [10,11]. Corticosteroids effectively reduce inflammation and pain, but often have to be administered on repeated occasions to sustain their effect. Moreover, corticosteroids are sometimes disputed, since they potentially have a detrimental effect on the already compromised cartilage metabolism [5,11]. In addition, they are most effective when there is a strong inflammatory component and less when the main problem is tissue degradation.

Hyaluronic acid has been reported to reduce inflammation, especially when administered intravenously. However, when administered intra-articularly, this effect is far less pronounced and it has been reported to cause flare reactions [11]. Because of these flare reactions, hyaluronic acid is often combined in practice with corticosteroid for intra-articular administration. A recent study on intra-articular use of hyaluronic acid has, however, reported no benefit of using hyaluronic acid combined with triamcinolone over triamcinolone alone [12].

Glucosamine and chondroitin sulfate are available as nutraceuticals and are popular in equine practice [13]. However, since nutraceuticals are not regulated, they are not standardly tested on safety and efficacy, and often information on their oral bioavailability is lacking [8,11].

Thus, currently, most treatments focus on reducing the symptoms of degenerative joint disease, but not to prevent further degradation. More recently, in search for treatment alternatives, cell-based therapies are being investigated because of their biological nature and regenerative potential [14–17]. Indeed, there are indications that intra-articular application of mesenchymal stem cells (MSCs) improves cartilage healing [14–16]. In addition, in vitro observations have demonstrated that chondrogenic induced MSCs produce cartilage-specific substances, such as aggrecan, glycosaminoglycans, and collagen type II, which could aid cartilage repair [17–20].

Despite the mode of action of MSCs being largely undefined, several mechanisms observed in in vitro and animal experiments are assumed to be responsible for the effect seen in patients [21]. The classic hypothesis that MSCs migrate to the site of injury, integrate in the tissue, and differentiate into functional cells is being progressively abandoned. Currently, it is inferred that MSCs heal injured tissue by using paracrine signals, cell-cell contact through nanotubes, cell fusion events, or by the secretion of extracellular vesicles [21,22]. These mechanisms are deemed responsible for the anti-inflammatory and angiogenic effect MSCs display under certain circumstances and the reason they can stimulate local cell survival and proliferation [21,22].

MSC-based therapies would thus potentially provide a more durable solution to degenerative joint disease, since they can possibly stimulate local cartilage repair and thus retard or even reverse the disease process. However, because experimental models do not completely resemble clinical pathology of naturally occurring degenerative joint disease, veterinary patient studies using autologous or allogeneic MSCs have also been conducted. These studies report the safety and efficacy of stem cell treatments based on an average amount of horses that were able to return to work or to return to previous levels of performance, which varied from 76% to 86% depending on the affected joints [23,24]. Nevertheless, these studies were not blinded and did not include control groups.

Thus, although horse owners hesitate to participate in clinical trials with placebo treatments, it is imperative to perform double-blinded superiority (compared to a placebo) or noninferiority (compared to a registered medicinal treatment) clinical trials to evaluate a new therapy for its effectiveness.

Veterinary MSC treatments are defined as a veterinary medicinal product according to the pharmaceutical act of the EU [Art. 1 No. 2 Directive 2001/82/Ethics committee (EC)] [25] and are thus subjected to strict regulations. In human medicine, 1,052 novel clinical stem cell trials have been identified so far, but only 3.5% resulted in a successful marketing authorization [26], indicating the difficulty to demonstrate evidence-based efficacy and/or safety of a cell-based therapy.

To the authors' knowledge, in veterinary medicine, no clinical field trial for cell-based products in equine orthopedics has been reported to date. However, to prove safety and efficacy of a novel stem cell-based product in horses, a field trial should be conducted compliant to Good Clinical Practice (GCP), as described by the Veterinary International Conference on Harmonization (VICH) Guideline number 9 [Committee for Medicinal Products for Veterinary Use (CVMP)/VICH/595/98]. Agreed by EU, Japan, and the United States, this quality standard provides detailed guidance on the requirements of clinical studies needed to obtain marketing authorization of new veterinary medicinal products in these markets.

Thus, based on this guideline, a field trial should be controlled, double blinded, multicenter, and randomized to effectively evaluate efficacy and safety of a cell-based therapy for treatment of naturally occurring degenerative joint disease in horses.

Therefore, to accommodate current legislation and to address the scientific need for a more durable solution, a placebo-controlled, double-blinded, multicenter, randomized GCP-compliant clinical field trial was performed, evaluating the safety and efficacy of equine MSCs as a treatment for naturally occurring degenerative joint disease (or chronic joint inflammation as an early stage of degenerative joint disease) in horses.

Since it has been reported that the microenvironment in inflamed joints has an influence on the paracrine signaling of equine MSCs [27], the MSCs used in this study were chondrogenically primed to stimulate the cells to produce the correct paracrine substances. Moreover, equine allogeneic plasma (EAP) was added to the MSCs before injection, because this has been shown to increase clinical improvement of horses with degenerative joint disease of the fetlock [17].

The hypothesis of this study was that allogeneic chondrogenic induced MSCs (ciMSCs) combined with EAP would be a safe and effective treatment, and would have superior and clinically relevant outcome compared to a placebo (saline) for the treatment of mild-to-moderate (early) degenerative fetlock joint disease in 75 horses.

## Materials and Methods

### Regulatory requirements and animal welfare regulations

This study was carried out in accordance with recommendations of the Animal Welfare Department of the Belgian Federal Public Service of Health. The study protocol was approved (EC_2015_003) by the Ethics Committee of Global Stem cell Technology (Permit Number: LA1700607). In addition, the study was conducted according to European and national regulatory requirements and in compliance with Directive 2001/82/EC, VICH GL9 (GCP, June 2000), EMA/CVMP/Efficacy Working Party (EWP)/81976/2010 (Guideline on statistical principles for clinical trials for veterinary medicinal products).

The medicinal products in this trials were produced according to Good Manufacturing Practice (GMP) (certificates: BE/GMP/2015/082 and BE/GMP/2016/069) and with manufacturing authorization 1868V for veterinary medicinal products. The field study was approved and received clinical trial authorization permit 0002829 from the Belgian federal agency for medicines and health products. Before enrolment, written owner consent was obtained from each owner of horses participating in the study.

### Investigational veterinary product and control product

The investigational veterinary product (IVP) consisted out of a proprietary combination of allogeneic ciMSCs and EAP. Preparation of the IVP is briefly described below. Saline (0.9% NaCl = Vetivex 9 mg/mL; Dechra Limited, Staffordshire, United Kingdom) was used as the control product (CP).

#### Isolation and chondrogenic induction of MSCs

In total, 50 mL of blood was collected in sterile ethylenediaminetetraacetic acid (EDTA) tubes from the *vena jugularis* of a 6-year-old donor gelding, which was tested for 32 different transmittable diseases at Böse laboratory (Harsum, Germany), in agreement with the European Medicines Agency (EMA) requirements. This donor horse was further not involved in the study in any way. Approval of the ethics committee was obtained for blood sampling of the donor horse (EC_2012_001 and EC_2016_003).

Isolation, characterization, and freezing of the intermediate cell stock were performed at P5 as described previously [20]. Cells were thawed, cultured, and subsequently chondrogenically induced from P9 to P10 using a proprietary method and media. The cells were characterized by assessing the total cell number, viability, and sterility, gene expression of a chondrogenic marker (cartilage oligomeric protein: COMP), and the presence of cell surface markers [cluster of differentiation (CD45), major histocompatibility complex (MHC) II, CD29, CD44, and CD90].

Chondrogenic induced MSCs were trypsinized, resuspended in 1 mL of Dulbecco's modified Eagle's medium low glucose (DMEM LG) with 10% of dimethyl sulfoxide (Sigma) at a concentration of 2 × 10^6^ cells per mL, and frozen before being shipped on dry ice for clinical application. Viability and gene expression of COMP were again assessed after 6 and 12 months of frozen storage to assess ciMSC batch stability.

#### Preparation of EAP

In total, 900 mL of peripheral blood was collected from a single donor horse (a gelding of 14 years) in a citrate phosphate dextrose adenine-1 single blood bag (Terumo^®^) for EAP preparation. This donor horse was a different individual from the stem cell donor, but was also tested for 32 different transmittable diseases at Böse laboratory, in agreement with the EMA requirements. This donor horse was also further not involved in the study in any way.

One hundred samples of 1 mL EAP were prepared as previously described by our group [28,29]. Each sample contained ∼85 × 10^6^ platelets and was frozen and stored at −80°C until clinical application.

### Patient inclusion and exclusion criteria

In total, 75 warmblood horses, 3 to 23 years of age, with recurrent lameness were enrolled in this study: 22 mares, 16 geldings, and 37 stallions. The MHC status of each individual patient was not determined. However, horses were not expected to be MHC matched with our MSC donor. Thus, most likely, horses were semiallogeneic or full allogeneic for MHC molecules.

To be included, horses had to present grade 2 or 3 lameness on the American Association of Equine Practitioners (AAEP) scale associated with (early staged) fetlock degenerative joint disease lasting for at least 2 months (early staged degenerative joint disease was defined as joint inflammation lasting over 2 months). In addition, lameness had to be confirmed by a positive intra-articular anesthesia of the fetlock and a positive flexion test. The fetlock joint also needed to show at least one sign of inflammation (swelling, pain on palpation, or heat assessed by palpation).

Horses were excluded if they received an unauthorized pretreatment (eg, corticosteroids), which still could have an effect on the pathology, if they had a severe medical condition that, in the opinion of the investigator, would have compromised their safe participation in the study, and if the horses had any condition, actual or anticipated, which the investigator felt would restrict or limit their successful participation for the entire duration of the study.

Other exclusion criteria consisted of previous participation in a stem cell study with the treated joint, lameness on more than one limb, AAEP scores of 1, 4, or 5, or lameness due to any other locomotion problem (nervous system and back problem). Moreover, a narrowed joint interspace reducing ≥1/3 of the normal fetlock joint space on lateromedial (LM) or dorsoplantar (DP) X-ray was not allowed. All horses were withdrawn from medication from study start to study completion. In addition, no therapies were offered at the study end.

### Blinding and randomization

Due to the color difference between the IVP and the CP, the study was blinded by using separate personnel for clinical examinations (investigator and examining veterinarian) and administration of treatments (dispenser); so there were two separate teams (examining vet and dispenser) at both study sites (two veterinary clinics) performing the different tasks within the study. Owners were not present when the treatment was administrated, so they were also blinded to which treatment their horse had received. A random treatment allocation plan was prepared for each study site and provided separately to the dispenser. The investigator/examining veterinarian assigned a unique ID number to the animal. The random treatment allocation plan was created using a block size of 3, whereby a ratio of 2:1 horses (IVP:CP) was allocated to the treatment groups.

### Treatment and rehabilitation protocol

On day 0, all inclusion and exclusion criteria were evaluated, an intra-articular anesthesia was performed on the affected joint, and LM and DP X-rays were taken. The horses were treated ∼24 h later (day 1) to avoid mixing between anesthetics and equine MSCs in the joint, which could cause severe cell damage [30]. On day 1, each horse was sedated using detomidine hydrochloride (Detonervin 10 mg/mL, 0.5 mL IV; Le Vet B.V., The Netherlands), and ketoprofen (Ketofen 10%; Merial Animal Health, Belgium, 10 mL IV) was given as concomitant nonsteroidal anti-inflammatory treatment before intra-articular application of 2 mL of the IVP or 2 mL of the CP. For the IVP administration, both the EAP (1 mL) and ciMSCs (1 mL) were thawed at 25°C–37°C and drawn into one syringe. The IVP was injected immediately after mixing.

The first 3 days of the study, the horses were walked up and down a corridor only. After that, they were walked with the rider up to 1 week, followed by walking and trotting with the rider up to the first clinical evaluation at week 3. Depending on the clinical status of the horse, they gradually returned to work by including canter exercises on week 4 and return to full work at week 6 or continued restricted walking and trotting exercises up to week 6. The rehabilitation occurred in the owners care or at the veterinary clinic the first 3 days of the study. The remainder of the study, the rehabilitation occurred in the owners care.

### Evaluation protocol

On day 0 and at week 3 and 6, a visual lameness assessment and flexion test were performed on all horses included in the study, using the AAEP score system and the score system depicted in Table 1, respectively. On day 1 and 2, a reduced lameness examination was performed by walking the horse up and down a corridor and evaluating lameness based on the following scores:

- no lameness visible (score 0),
- lameness difficult to observe and not consistent (score 1)
- lameness obvious at walk (score 2)
- minimal weight bearing (score 3).

**Table 1.** Overview of the Score Systems Used by the Examining Veterinarians for Assessing Lameness, Response to Flexion Test, Joint Effusion, Heat at Palpation, and Pain to Pressure, and the Scores Attributed by the Owners During the Owner Questionnaires

*Parameter*	*Score*	*Clinical implication*
*Veterinary scoring*		
AAEP grading	0	No lameness
	1	Lameness not consistent, regardless of circumstances
	2	Lameness consistent under certain circumstances
	3	Lameness consistently observable on a straight line
	4	Obvious lameness: marked nodding or shortened stride
	5	Minimal weight-bearing lameness in motion or at rest
Flexion test	0	No flexion response
	1	Mild flexion response
	2	Moderate flexion response
	3	Severe flexion response
Joint effusion	0	No swelling
	1	Mild swelling
	2	Moderate swelling
	3	Severe swelling
	4	Extreme swelling (also periarticular)
Heat at palpation	0	No increased temperature sensation
	1	Mild increased temperature sensation
	2	Moderate increased temperature sensation
	3	Severe increased temperature sensation
Pain to pressure	0	No pain to pressure
	1	Mild pain to pressure
	2	Moderate pain to pressure
	3	Severe pain to pressure

*Owner scoring*		
Horse improvement	0%	Not at all
	20%	Marginal improvement
	40%	Mild improvement
	60%	Moderate improvement
	80%	Remarkable improvement
	100%	No more discomfort noticeable
Current working status	00	Failure to return to work
	0	Rehabilitating
	1	Return to work
	2	Return to previous level of work

AAEP, American Association of Equine Practitioners.

Based on other clinical studies [17,23], week 6 was defined as the time point to evaluate the primary efficacy endpoint to observe a sustained clinical effect of treatment. A relevant clinical improvement, the primary efficacy criterion, was considered a reduction of AAEP lameness score from 2 or 3 (clear clinical lameness) at inclusion to an AAEP score of 0 or 1 (no or inconsistent clinical lameness). The total duration of the study per animal was allowed to range from 37 to 47 days (day 0 to week 6 ± 5 days). At week 6, the owners were also consulted to rate the condition and improvement of their horse (Table 1). Further veterinary evaluation (joint and lameness assessment scored according to Table 1) was planned for week 12 ± 1 week and week 18 ± 1 week. In case the patient could not be presented to the investigator, an owner questionnaire was used to obtain data from the patient. In addition, at week 18 and 1 year after treatment, owners were contacted again to inform about the work status of their horse.

Any observation in animals that was unfavorable and unintended, and occurred after the use of the IVP or CP was defined an adverse event (AE). A suspected adverse drug reaction was defined as an AE where a relation to treatment was suspected. A serious adverse event (SAE) was defined as any AE that resulted in death, was life-threatening, or resulted in persistent or significant disability/incapacity. As lameness and joint abnormalities in the treated affected limb were recorded separately as they were part of the efficacy evaluation criteria, these were not documented separately as AEs.

A clinical examination was performed of each horse on day 0, 1, and 2 and at week 3 and 6, and whenever requested by the owner, and consisted of temperature, respiratory rate, and heart rate measurements combined with a general body examination. Local clinical inflammatory parameters, such as heat at palpation, pain to pressure, and joint swelling were also scored at these time points (Table 1).

### Statistical analysis

The sample size of the study was calculated using SAS^®^ statistical analysis software (Version 9.3) of the SAS Institute, Inc. (Cary, NC), so a two-group χ^2^ test with a 0.05 two-sided significance would have 80% power to detect the difference between a control proportion, π_1_, of 0.3 (30% estimated success) and an IVP proportion, π_2_, of 0.6 or 0.7 (60%–70% estimated success). Based on this calculation with an unbalanced distribution of group (IVP:CP = 2:1), at least 50 animals in the IVP treatment group and 25 animals in the CP per-treatment group were shown to be sufficient to demonstrate potential statistical superiority.

All data were collected on pre-established data capture forms. Then data were entered to a specifically established electronic database, verified, and inconsistencies sorted. Statistical analysis was performed on the data transferred from that database to SAS statistical analysis software (Version 9.3) of the SAS Institute, Inc. The difference in relevant clinical improvement scores was compared between groups at different time points using Fisher's exact test. To compare all clinical and owner scores presenting number and percent of each score category, the Mantel–Haenszel test was used. The difference in working status was compared using Mann–Whitney *U* test. The percentage of animals with AEs was compared between groups using Fisher's exact test. A 5% level of significance was used to assess statistical differences.

## Results

### Isolation and characterization of MSCs

**FIG. 1.**
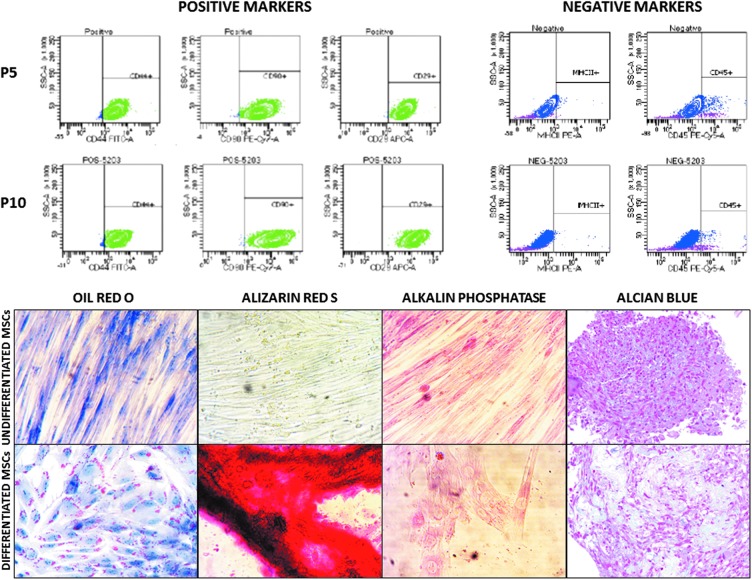
Representative flow cytometric images of positive (CD29, CD44, and CD90) and negative (CD45 and MHC II) MSC markers. Adipogenic differentiation was confirmed with Oil Red O staining, osteogenic differentiation with alizarin red S and alkaline phosphatase staining, and chondrogenic differentiation with alcian blue staining. Differentiated cells demonstrated morphological changes and positive staining areas, whereas undifferentiated cells remained spindle shaped without convincing staining. CD, cluster of differentiation; MHC, major histocompatibility complex; MSC, mesenchymal stem cell.

The intermediate cell stock displayed all properties to be characterized as MSCs. Briefly, they attached to plastic, trilineage differentiation was performed successfully, and MSCs were positive for CD29 (100%), CD44 (100%), and CD90 (100%) and negative for CD45 (1%) and MHC II (0%) (Fig. 1). The average population doubling time over 10 passages was 1.4 and passage 10 ciMSCs displayed 96% viability, an MSC immunophenotype (100% CD29, 87% CD44, 98% CD90, 2% CD45, and 0% MHC II), and a 4.4-fold COMP increase as a marker for chondrogenic induction [17]. After 6 months of frozen storage, 82% viability and 4.6-fold COMP change were present. At 12 months, viability remained above 80% and a 4.2-fold COMP change was present. All horses were treated within this period after MSC production.

### Clinical outcome

**FIG. 2.**
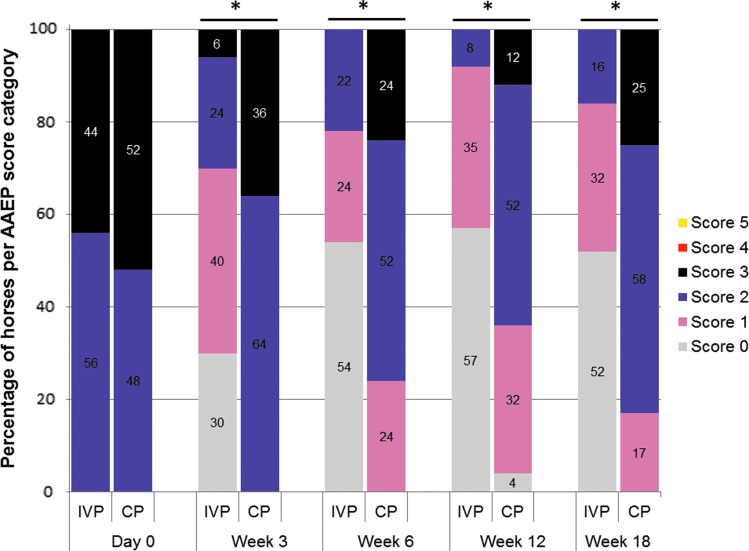
The distribution of horses (in percentages) over the AAEP score categories per time point for the IVP-treated group and placebo control (CP)-treated group. The asterisk (*) indicates a significant difference in frequency of scores between the two treatments groups (*P* < 0.001). AAEP, American Association of Equine Practitioners; CP, control product; IVP, investigational veterinary product.

**FIG. 3.**
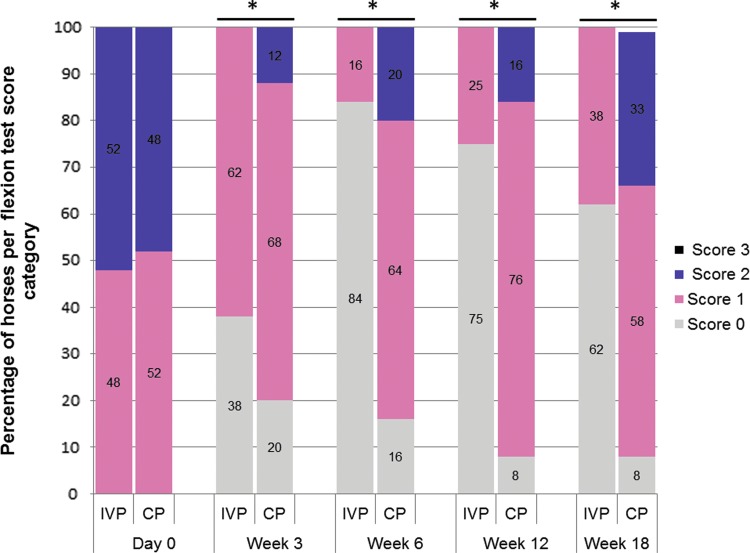
The evolution of the percentage of horses per flexion test score category over time for the IVP-treated group and placebo control (CP)-treated group. The asterisk (*) indicates a significant difference in frequency of scores between the two treatments groups (*P* < 0.05 for week 3 and *P* < 0.001 for weeks 6, 12, and 18, respectively).

**FIG. 4.**
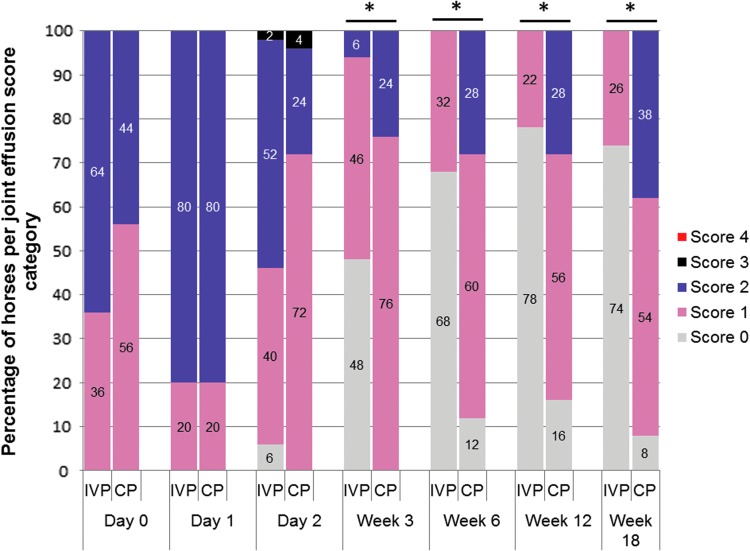
The distribution of horses (in percentages) over the joint effusion score categories per time point for the IVP-treated group and placebo control (CP)-treated group. The asterisk (*) indicates a significant difference in frequency of scores between the two treatments groups (*P* < 0.001).

All 75 horses included in this study showed initially a moderate lameness (score 2–3 out of 5 on the AAEP scale; Fig. 2), mild-to-moderate response to flexion test (score 1–2 out of 3; Fig. 3), and mild-to-moderate joint swelling (score 1–2 out of 4; Fig. 4). The clinical signs in both treatment groups upon inclusion were comparable and did not show any significant difference between groups (Table 2).

**Table 2.** Evaluation of Inclusion Criteria for Both Treatment Groups

*Baseline parameters*	*IVP (*N* = 50)*	*CP (*N* = 25)*	*Comparison* P *value*
Age in years			
Mean (SD)	9.9 (3.42)	10.1 (4.81)	0.702^a^
Min–max	3–17	5–23	
Median	10.0	9.0	
Q1–Q3	7.0–12.0	6.0–12.0	
Gender, *n* (%)			
Gelding	11 (22)	5 (20)	0.152^b^
Mare	18 (36)	4 (16)	
Stallion	21 (42)	16 (64)	
AAEP score, *n* (%)			
2 = lameness difficult to observe	28 (56)	12 (48)	0.516^c^
3 = lameness consistently observable	22 (44)	13 (52)	
Flexion score, *n* (%)			
1 = mild response	24 (48)	13 (52)	0.746^c^
2 = moderate response	26 (52)	12 (48)	
Pain score, *n* (%)			
0 = no pain to pressure	37 (74)	16 (64)	0.373^c^
1 = mild pain to pressure	13 (26)	9 (36)	
Heat score, *n* (%)			
0 = no increased temperature	31 (62)	16 (64)	0.867^c^
1 = mild increased temperature	19 (38)	9 (36)	
Swelling score, *n* (%)			
1 = mild swelling	18 (36)	14 (56)	0.101^c^
2 = moderate swelling	32 (64)	11 (44)	

^a^ *P* values are based on the Wilcoxon rank-sum statistic.

^b^ *P* value is based on the Fisher's exact test.

^c^ *P* values are based on the Mantel–Haenszel chi-square statistic.

AAEP, American Association of Equine Practitioners; CP, control product; IVP, investigational veterinary product; Q, Quartile; SD, standard deviation.

At week 3 after treatment, the AAEP lameness score was significantly (*P* < 0.001) improved in the IVP group compared to the CP group with relevant clinical improvement (decrease to AAEP score 0 or 1) in 70% of the animals (Fig. 2). At week 6, relevant clinical improvement as a primary efficacy criterion was observed in 78% of the IVP-treated horses in comparison to 24% in the CP-treated horses (Fig. 2). This difference of 54.0% was significant, and superiority was shown for IVP compared to CP (*P* < 0.001). The evaluation of the AAEP lameness scores at week 12 (92% vs. 36%) and 18 (84% vs. 17%) statistically (*P* < 0.001) confirmed a long-term clinical improvement in the IVP group when compared to the CP group (Fig. 2). In the CP-treated group, 64% of the animals still showed a lameness score of 2 or 3 at week 12 and 83% at week 18 (Fig. 2). The onset of a clinically relevant improvement with the IVP treatment was shown as early as week 3, and this effect continued until week 18.

The distribution of scores of the secondary efficacy criterion flexion test response and joint swelling was significantly different between groups at all postbaseline examinations, week 3, 6, 12, and 18, with lower scores in the IVP-treated animals than in CP-treated animals (Figs. 3 and 4). At week 3, no flexion response was observed in 38% of IVP-treated horses, which was significantly higher than the 20% of CP-treated horses (*P* < 0.05) (Fig. 3). In the CP group, the percentage of animals with a negative response to flexion decreased over time toward 8% of the horses at week 12 and 18 (Fig. 3). In the IVP-treated group, the percentage of negative flexion response was 84% at week 6 and reduced to 75% at week 12 and 62% at week 18, which was significantly better than in the animals of the CP group at all time points (*P* < 0.001) (Fig. 3).

**FIG. 5.**
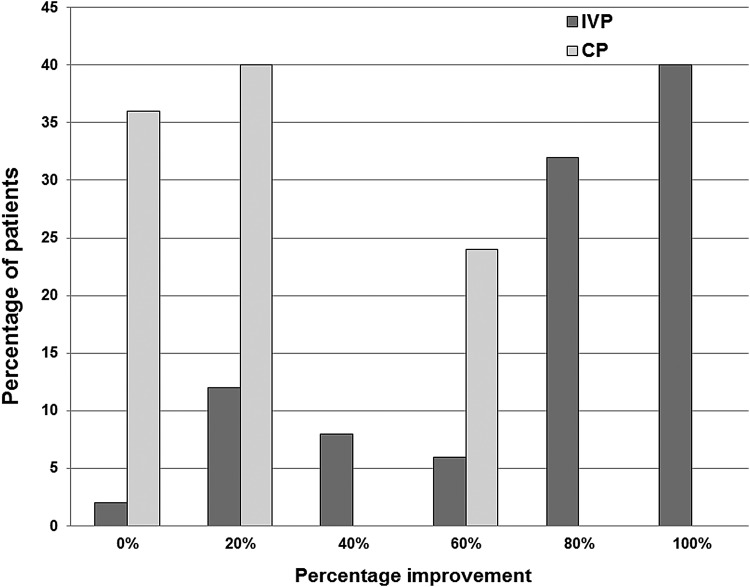
The distribution of the horses (in percentages) over the clinical improvement categories indicated by the owners at week 6 for both treatment groups. There was a significant difference (*P* < 0.001) between the IVP and placebo control (CP) group for each score category.

At week 6, owners rated at least an 80% improvement in 72% of the IVP-treated animals. This was significantly more (*P* < 0.001) than for the CP-treated horses (Table 3 and Fig. 5). At week 12 and 18, one horse of the IVP and CP group, respectively, was not presented for follow-up examination. The owner questionnaire indicated that one of these animals returned to work (IVP treatment) and the other animal was still rehabilitating (CP group).

**Table 3.** Distribution of Horse Improvement Scores as Indicated by the Owners at Week 6 After Treatment

*Score*	*IVP (*N* = 50)*	*CP (*N* = 25)*	*Total (*N* = 75)*	*Comparison* P *value*
*Horse improvement*				
0%	1 (2%)	9 (36%)	10 (13%)	<0.001
20%	6 (12%)	10 (40%)	16 (21%)	
40%	4 (8%)	0 (0%)	4 (5%)	
60%	3 (6%)	6 (24%)	9 (12%)	
80%	16 (32%)	0 (0%)	16 (21%)	
100%	20 (40%)	0 (0%)	20 (27%)	

**FIG. 6.**
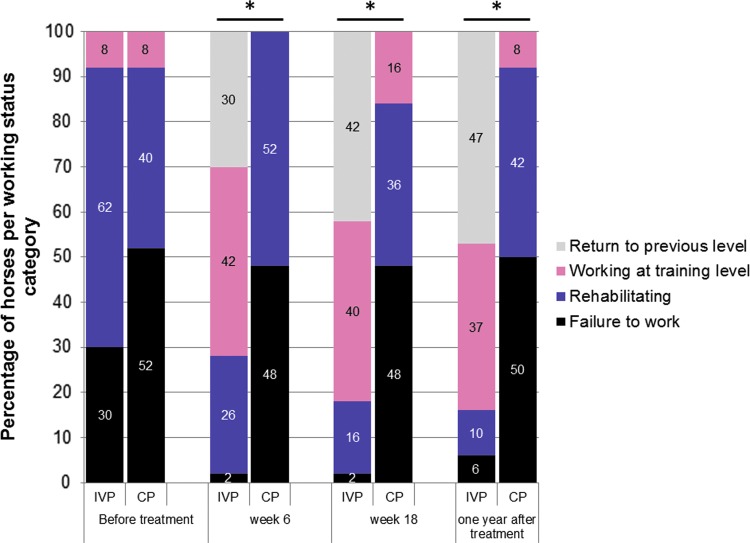
The distribution of the horses (in percentages) over the working status categories indicated by the owner per time point for the IVP-treated group and placebo control (CP)-treated group. The asterisk (*) indicates a significant difference in frequency of scores between the two treatments groups (*P* < 0.001).

There was a significant difference in working status of animals between the IVP and CP group (*P* < 0.001) at every time point after baseline (Fig. 6). At week 6, 30% of the horses in the IVP group already returned to their previous level of work compared to none of the horses in the CP group. In addition, 42% of the horses in the IVP group were working at training level compared to none of the CP group horses. At week 18, the percentage of horses that returned to their previous level of work increased to 42% in the IVP group, while in the CP group, this was still 0% (Fig. 6). Therefore, at week 18, in the IVP group, 82% of the horses returned to some level of work (working at training level+returned to previous level) compared to 16% in the CP group. One year after treatment, in the IVP group, 37% of the horses were working at training level compared to 8% of the horses treated with CP. Moreover, 47% of the horses treated with IVP returned to their previous level of work, compared to none of the horses treated with CP (Fig. 6).

Concomitant medication after study completion (week 6), that is, a single intravenous NSAID injection, was necessary for two animals (4%) in the IVP group and for nine animals (36%) in the CP group, which was statistically significant (*P* < 0.001), yet did not substantially influence the later scorings of these animals at week 12 and 18.

In total, three AEs were observed in 3 animals out of 75 allocated to treatment, all diagnosed as mild infections of the upper respiratory tract showing nasal discharge. Two of the AEs were observed in the IVP group and one in the CP group (4.0% in both groups) with no statistically significant difference between groups (*P* = 1.000). Neither of the AEs was serious nor regarded as related to the study treatment.

At the reduced lameness assessment on day 2, no statistical significant difference between groups was observed (*P* = 1.000).

There was no significant difference between groups in the frequency of local reactions (joint effusion, heat at palpation, and pain to pressure) within the first 2 days after treatment (*P* > 0.999). In one animal (2%) in the IVP group, joint swelling and heat at the injection site worsened from day 1 to 2 by one score point and pain to pressure worsened in another animal (2%) from day 1 to 2 by one score point. In the CP group, increased joint swelling was observed in one animal (4.0%) from day 1 to 2. From week 3 to 18 onward, animals in the IVP group demonstrated significantly (*P* < 0.001) less joint effusion, of which 68%–78% of the horses were without joint swelling from week 6 onward in comparison to 49%–57% in the CP group (Fig. 4).

The presence of pain to pressure at the injection site was not significantly different at observations at week 3 and 6 (*P* ≥ 0.071) with the majority of animals in both groups (ranging from 88.0% to 100%) showing no local pain to pressure. Although significantly lower scores were observed in the IVP group than in the CP group at weeks 12 and 18 (*P* < 0.015), the majority of animals (ranging from 87.5% to 100%) of both groups showed no local pain to pressure at these observations. Except for week 6 (*P* < 0.05), the presence of heat at the injection site was not significantly different between groups on any postbaseline day (*P* > 0.149). However, the majority of animals in both groups (ranging from 95.8% to 100%) showed normal temperature at the injection site.

## Discussion

In this study, 75 horses with naturally occurring fetlock joint inflammation were enrolled, of which 50 horses were treated with allogeneic chondrogenic induced MSCs combined with EAP (IVP) and 25 with a placebo, being 0.9% NaCl (CP). Such negative controlled study tends to be a challenge, especially to convince owners to participate, but was necessary due to the lack of a licensed treatment suitable for comparison in this study. In this study, exit criteria had been defined to allow withdrawal of animals from the study in case of relevant pain, so owners were reassured their horse would not have to suffer from unnecessary pain if enrolled in the study. An ethical committee had reviewed the study protocol before implementation.

Relevant clinical improvement and effusion scores increased or remained stable in the IVP-treated group from week 3 toward week 18, whereas the opposite trend was present in the CP-treated horses. Interestingly, 20%–25% of the saline-treated horses also demonstrated relevant clinical improvement at a certain time point. This could be due to the adjusted training program or to the therapeutic effect of saline as CP, which has been reported previously [31]. Nevertheless, for all observed parameters, clinical improvement decreased over time in the CP group. In the IVP group, the negative joint flexion also decreased from 84% of the horses at week 6 to 62% of the horses at week 18. However, at the long-term follow-up of 1 year, 84% of the horses in the IVP group were working at training level or their previous level, indicating a long-term sustainability of the IVP-treated joints.

In this study, EAP from a single donor horse was added to the ciMSCs, which is meant to improve MSC proliferation and chondrogenic differentiation [32]. Indeed, when using EAP, the manufacturing process needs to be well controlled and standardized to produce a reproducible product and to reduce white and red blood cells as much as possible to prevent transfusion-related erythrolysis. In this study, a single injection of allogeneic EAP and MSCs was performed and did not cause any sign of product-related AEs. Nasal discharge was observed in some animals of both groups, indicating typical seasonal disease during the fall and winter months. They were considered not to be related to the treatment.

In general, allogeneic MSCs are being considered equally immunomodulating as autologous MSCs in vitro [33,34] and in vivo [35,36], but it has been reported that repeated intra-articular administration of allogeneic equine MSCs may cause a significant increase in total nucleated cell count [37]. This increase was still within acceptable clinical ranges [38] and another study reported no AEs after repeated injection of pooled allogeneic MSCs [39]. However, it should be considered that MSCs are heterogeneous in MHC II expression [40], and should therefore be tested in vitro before clinical application, as reported in this study. Moreover, certain inflammatory parameters can upregulate MHC molecules, which increases the chances for graft rejection [40,41]. This could also explain the increase in total nucleated cell count as mentioned above [37].

In this regard, a recent study reported a reduced MHC expression after pretreating MSCs with transforming growth factor-β (TGF-β) [42]. Since this happens to be one of the cartilage stimulating growth factors used in this study to chondrogenically predifferentiate the MSCs, it might explain why a very low number of horses presented with clinical signs of inflammation after the injection. Moreover, the MHC I level on peripheral blood-derived MSCs is below 10% [17], offering another explanation for the low number of horses with clinical signs of inflammation in this study.

The patients in this study were also not matched with the donor MSCs and since no clinical problems were detected, this further confirms the low immunogenicity of the donor cells. Nevertheless, it should be mentioned that all horses in this study received a single injection of ketoprofen (NSAID) on the day of intra-articular injection, which may have masked a potential increase of nucleated cells; horses were injected only once with the IVP. Further research with repeated injections using the IVP without NSAID administration should provide more insights in this matter and determine whether the same safety and efficacy can be observed after repeated injections. It would also be interesting to evaluate antibody response in future studies and determine whether the same clinical results would be achieved with another donor horse (with planned donor/acceptor mismatch) or another dose.

Another important and underestimated aspect of the use of regenerative medicinal products is the use of an optimal dose. Consideration of the lowest effective dose for allogeneic MSC therapy is of highest importance, because high doses (30–50 million) of allogeneic equine MSCs could induce antibody responses in vivo [43]. Based on previous clinical studies to evaluate the effect of allogeneic ciMSCs [17,23], the dose of ciMSCs used in the IVP in this study was set at 2 million cells, which is 5–25-fold lower than the reported 10–50 million MSCs used in other equine studies [15,24,37,43,44]. Similarly, it has been described in dogs that higher treatment doses (66 million) of allogeneic MSCs result in lameness and pain, whereas this was not the case with lower doses (5 million) [45].

Even though dose-dependent effects have been reported for allogeneic MSCs for the treatment of myocardial infarction in rats [46] and graft versus host disease in mice [47], others have also demonstrated a superior outcome using a lower dose of allogeneic MSCs for treatment of injured medial collateral ligaments of rat knees [48] or for the treatment of human knee osteoarthritis [49]. In addition, after an intravenous injection of allogeneic adipose-derived MSCs in cats with chronic kidney disease, dose-related adverse effects were observed [50]. After reducing the dose from 4 to 2 million MSCs per mL, limited adverse effects and clinically interesting results [51] were noticed by the authors. All these findings confirm that the selection of the dose of allogeneic MSCs needs careful consideration.

In this study, no treatment group was included that only received the excipient EAP. However, no animals were treated with EAP alone to reduce the number and use of animals. Moreover, The IVP consists of a proprietary combination of allogeneic ciMSCs and EAP. Thus, horses would never be treated with EAP alone. In addition, the main contribution of EAP to the ciMSCs is a significant increase in cell viability after thawing of frozen ciMSCs (3%–8% increase in viability depending on storage duration of the ciMSCs *P* < 0.05: Data not shown). Because the thrombocyte specifications of EAP are within physiological range (75,000–300,000/μL) [52], no influence of the EAP on the healing process is expected.

On the other hand, when producing actual platelet-rich-plasma (PRP) with platelet levels more than double of the physiological platelet levels, a short-term significant increase in white blood cell counts, prostaglandin E2, and total protein concentrations was detectable in synovial fluid analysis within 6–48 h after injection into equine fetlock joints [53]. In the proof-of-concept study performed with the IVP (including EAP), no such findings were visible at any of the reported time points (unpub. obsvns.). Moreover, in a previous study, we demonstrated that ciMSCs in combination with allogeneic plasma result in significant increased clinical outcomes in comparison to allogeneic plasma treatment alone in fetlock joints [17].

Lameness was evaluated using visual assessment and the AAEP scoring system and not by an objective measuring tool such as a lameness locator. However, the use of this AAEP score system was just, since Keegan et al. [54] stated, “Such objective measures may augment, but not replace results obtained by subjective evaluation of lameness in horses.” Although the lameness locator system demonstrated to have a better interobserver repeatability than a subjective lameness examination [55,56], the system is still flawed and not accepted as the gold standard for lameness evaluation in the literature [54,55]. In addition, the lameness locator system only takes into account vertical head and pelvis movement and leaves out several different other kinematic parameters, which can indicate lameness (eg, decreased maximum fetlock extension and decreased limb retraction or protraction).

In the subjective lameness examination, however, the examining veterinarian can take all these parameters into account. Moreover, studies on the repeatability of subjective lameness examination are often based on assessment of video tapes without sound and a shot from only one angle and assessing only one circumstance, while it has been demonstrated that a full live lameness examination improves interobserver agreement of a subjective lameness examination [55,57–59].

However, recognizing the limitations of a subjective lameness examination, some preventive measures were taken to increase objectivity: (1) horses were only included if they presented with an initial lameness score of 2 or 3 on the AAEP scale (consistent lameness), (2) lameness was confirmed for that limb by intra-articular anesthesia and a positive flexion test, (3) the examiner was blinded for treatment, but not for the initial side of lameness, facilitating lameness detection for that particular limb, (4) subjective examination was performed in exactly the same way for placebo-treated and IVP-treated horses, so any bias generated during the subjective lameness examination would have been the same for the two treatment groups, and (5) all lameness examination was performed by experienced veterinarians.

In conclusion, our results indicate that 2 million allogeneic chondrogenic induced MSCs with EAP administered once in the joint has a similar safety profile and superior efficacy compared to a placebo in the treatment of inflammatory fetlock joint disease in horses. Indeed, besides nasal discharge in both treatment groups, no AEs were observed during the entire study period. Moreover, the effect of the IVP was proven for the duration of 18 weeks based on relevant clinical improvement, namely a decrease in lameness scores, a decrease in response to flexion, and a decrease in joint effusion. Furthermore, the effect of the treatment was confirmed to sustain 1 year after administration, with significantly more horses working at their previous level or at training level at this point compared to the placebo control group.
